# A novel German guideline for the sampling procedures for passive biomonitoring with fish as accumulation indicators: VDI 4230, Part 4

**DOI:** 10.1186/s12302-016-0081-x

**Published:** 2016-05-04

**Authors:** Roland Klein, Jens Gercken, Herbert Löffler, Theo von der Trenck, Thomas Braunbeck

**Affiliations:** 1 Department of Biogeography, University of Trier, Universitätsring 15, 54286 Trier, Germany; 2 Institute of Applied Ecology, Alte Dorfstraße 11, 18184 Neu Broderstorf, Germany; 3 Institute for Lake Research, State Institute for the Environment, Measurements and Conservation in Baden Württemberg, Argenweg 50/1, 88085 Langenargen, Germany; 4 State Institute for the Environment, Measurements and Conservation in Baden Württemberg, Griesbachstr. 1, 76185 Karlsruhe, Germany; 5 Aquatic Ecology and Toxicology Group, Center for Organismal Studies, University of Heidelberg, Im Neuenheimer Feld 504, 69120 Heidelberg, Germany

## Abstract

A novel German guideline, VDI 4230, Part 4, has been adopted to provide a standardized protocol for the sampling of freshwater fish for passive biomonitoring with fish as accumulation indicators. The guideline has been designed for multiple purposes, e.g. for the chronological tracking of environmental pollution following hazardous incidents, for the monitoring of the success of regulatory measures and voluntary restrictions, for emission and immission monitoring, for the monitoring of contaminated sites, for the provision of samples retained for long-term monitoring, and for initial exploratory studies for determination of pollution hotspots.

Biological methods are important tools in environmental risk assessment and pollution monitoring. Fish are especially well-suited indicators for aquatic ecosystems due to their longevity, their ability for spatial and temporal integration, as well as their availability in different water types. In contrast to non-vertebrate bioindicators, fish allow a more direct transfer of observations to humans. Thus, especially for many highly lipophilic compounds they may also serve as indicators for the risk of humans to accumulate contaminants via the food chain. For these reasons, the new national guideline VDI 4230, Part 4, addresses fish as indicators of accumulation. As part of a comprehensive VDI guideline series on bioindication in wildlife, it provides standardized protocols for the sampling of fish in biomonitoring.

Monitoring the environmental status and the definition of environmental quality standards and objectives play an increasingly important role, especially in environmental policy. With the adoption of the Water Framework Directive 2000/60/EC (WFD [1]), the European Union has taken great strides towards the protection of aquatic environments. The aim of this directive is to reach and guarantee a good ecological and chemical status of water bodies. In this context, the German Federal States’ Water Consortium (LAWA) specifically developed a concept for biota studies for the monitoring of priority substances [2] in compliance with the European Directive 2008/105/EC [3]. This biota concept is currently under revision to implement European Directive 2013/39/EU [4]. In contrast to the LAWA concept, the new VDI guideline provides a detailed set of operational procedures applicable to many additional cases in the context of biomonitoring.

The new VDI guideline has been developed on the basis of an existing guideline for sampling and sample processing by the Federal German Environmental Specimen Bank [5]. The new guideline integrates the contents of this guideline and develops it further for additional purposes and fish species.

A reliable assessment of surface waters based on biota data strongly depends upon the availability of high-quality samples. Hence, sampling procedures are of crucial importance, because sampling errors cannot be corrected or mitigated at a later stage. Standardization of sampling procedures is the most important tool to assure a high degree of reproducibility without losing representativity.

The new guideline meets these requirements: It provides comprehensive descriptions of the principles and implementations of procedures and also gives advice on documentation and quality assurance to avoid artefacts, to minimize the loss of biological information and to cope with the high variability of biological samples. The selection of target species according to well-defined criteria as well as the selection of target compartments are crucial steps in the sampling process. Their implementation is extensively described and encompasses many different steps and requirements. These include, for example, species identification, legal requirements and approvals, selection and number of individuals, sampling period, recommended equipment and cleaning procedures, sampling technique, and sample transport. Individual operational steps are not only specified in detail, but are also well-founded and accounted for. This provides potential users with the necessary insight to apply and to adjust the various procedures to their own specific requirements by providing standard descriptions for sampling procedures including planning, catching the fish, as well as sampling and handling of tissues. Further information and tools for high-quality sampling are provided in two appendices: Appendix I focuses on age determination of fish using gill covers (opercula) or further ossifications. Appendix II gives examples of data sheets for a comprehensive documentation of the sampling design and techniques, as well as fish data.

The guideline is limited to fish from freshwater habitats and their use as accumulation indicators. This covers a wide range of applications within the scope of environmental and pollutant monitoring. Areas of application include, for example, the chronological tracking of environmental pollution following hazardous incidents, monitoring of the success of regulatory measures and voluntary restrictions, emission and immission monitoring, monitoring of contaminated sites, provision of samples retained for long-term monitoring, and initial exploratory studies for determination of pollution hotspots.

Currently, the use of living organisms for biomonitoring is imperative to protect human and environmental health. Nonetheless, the long-term goal is to replace the use of animals by alternative procedures as far as possible. The European Directive on the protection of animals used for scientific purposes (Directive 2010/63/EU) provides a framework to balance this conflict. Since alternative approaches have not yet been standardized and validated in the field of environmental monitoring, such methods are not covered by the new guideline VDI 4230, Part 4.

Since the rules and provisions for use of fish as general effect indicators differ substantially, a separate guideline for standards in effect monitoring is under preparation (VDI 4230, Part 5).
